# Evaluation of the deposition and distribution of spray droplets in citrus orchards by plant protection drones

**DOI:** 10.3389/fpls.2023.1303669

**Published:** 2023-11-29

**Authors:** Yu Yan, Yubin Lan, Guobin Wang, Mujahid Hussain, Huizheng Wang, Xiaoqing Yu, Changfeng Shan, Baoju Wang, Cancan Song

**Affiliations:** ^1^ College of Agricultural Engineering and Food Science, Shandong University of Technology, Zibo, China; ^2^ Shandong University of Technology Sub-center, National Center for International Collaboration Research on Precision Agricultural Aviation, Zibo, China; ^3^ Plant Protection Station of Shandong Province, Jinan, China

**Keywords:** plant protection drones, citrus, application volume, number of droplets, spray coverage

## Abstract

Plant protection drone spraying technology is widely used to prevent and control crop diseases and pests due to its advantages of being unaffected by crop growth patterns and terrain restrictions, high operational efficiency, and low labor requirements. The operational parameters of plant protection drones significantly impact the distribution of spray droplets, thereby affecting pesticide utilization. In this study, a field experiment was conducted to determine the working modes of two representative plant protection drones and an electric backpack sprayer as a control to explore the characteristics of droplet deposition with different spray volumes in the citrus canopy. The results showed that the spraying volume significantly affected the number of droplets and the spray coverage. The number of droplets and the spray coverage area on the leaf surface were significantly increased by increasing the spray volume from 60 L/ha to 120 L/ha in plant protection drones. Particularly for the DJI T30, the mid-lower canopy showed a spray coverage increase of 52.5%. The droplet density demonstrated the most significant variations in the lower inner canopy, ranging from 18.7 droplets/cm^2^ to 41.7 droplets/cm^2^ by XAG V40. From the deposition distribution on fruit trees, the plant protection drones exhibit good penetration ability, as the droplets can achieve a relatively even distribution in different canopy layers of citrus trees. The droplet distribution uniformity inside the canopy is similar for XAG V40 and DJI T30, with a variation coefficient of approximately 50%-100%. Compared to the plant protection drones, the knapsack electric sprayer is suitable for pest and disease control in the mid-lower canopy, but they face challenges of insufficient deposition capability in the upper canopy and overall poor spray uniformity. The distribution of deposition determined in this study provides data support for the selection of spraying agents for fruit trees by plant protection drones and for the control of different pests and diseases.

## 1 Introduction

Citrus is one of the top fruit tree species in the world, is incredibly vitamin-rich, has significant economic and therapeutic significance, and has overtaken apples as the most popular fruit in China. Citrus plants in China accounted for about a fifth of all garden fruit plantings in 2022, according to the list of China’s beneficial agricultural products (Kui and Qi, 2022). In the production activities of citrus cultivation, the reduction of citrus yield caused by infection with citrus pests and diseases is the main factor limiting the safe production of citrus (Glienke et al., 2011; Brentu et al., 2012; Sun, 2022). Regular pest and disease control is required to ensure crop yield and quality. As citrus planting areas are mostly on mountainous slopes with complicated road conditions, it is not convenient for ground mechanical spraying, which leads to a knapsack electric sprayer, which is still the main way of pesticide spraying. However, this spraying method not only requires a lot of human and material resources but also has a low efficiency, which causes an increase in planting costs and is not conducive to the development of the citrus industry. Therefore, growers need to use modern and efficient plant protection machinery to replace traditional spraying equipment (Yamane and Miyazaki, 2017).

In recent years, the rapid development of aerial spraying by plant protection drones has attracted attention and has been widely promoted and applied (Lan et al., 2017; Yang et al., 2018; Wang et al., 2019). Plant protection drones get rid of the ground operation mode, break through the restrictions of crop types (low and high crops, etc.), and have the advantages of high operational efficiency, low cost, good application effect, and strong ability to deal with sudden disasters (Xue et al., 2014). With the development and application of plant protection drones, plant protection drone flight operations have begun to extend from field crops to fruit trees, and relevant researchers are exploring the effects of different operating parameters of plant protection drones on the distribution of the number of droplets. Citrus canopy is relatively simple and mostly planted in patches and scales, which is more suitable for plant protection drones to operate. In 2016, Zhang et al. (2016) explored the spraying effect of a small electric quadrotor plant protection drones on the canopy of citrus trees at different flight heights and found that the plant protection drones deposited best when working on open-centered shaped plants at 1.0 m for height. Tang et al. (2018) studied the effects of operating height and tree shape on sap droplet deposition in citrus trees using plant protection drones. When the operation height was 1.2 m and the flight speed was 3.5 m/s, the number of droplets and the spray coverage in the canopy reached the maximum. Guo et al. (2022) evaluated four types of nozzles (SX110015, XR80015, IDK90015, and TR80015) based on a point spray pattern in a southern pear orchard experiment and showed that the IDK90015 nozzle showed significantly higher deposition and penetration. Several studies on fruit trees have compared plant protection drone applications with conventional application equipment. Martinez-Guanter et al. (2020) found that conventional shower applications cause excessive waste and that treatments using plant protection drones are more uniform while significantly reducing drift. There are still many barriers to using plant protection drones in citrus orchards, and in terms of plant protection drone technology, a combination of electrostatic nozzles and spray aids can improve droplet deposition. In addition, field testing requires attention to suitable weather conditions, especially wind speed. Zhang et al (Pan et al., 2017). 2017 verified that plant protection drones are twice as efficient as manual applications using field trials. Junior et al. (2016) studied water use and application rates for the control of citrus black spot and found that a change in spray volume from 125 mL/m3 to 75 mL/m3 resulted in a 40% reduction in CBS spray cost and water use and an increase in profit of up to 35%. Li et al. (2021) used an electric hexacopter plant protection drone to compare the overall residue levels of chlorothalonil insecticide at application rates of 46.8 L/ha and 93.5 L/ha in an almond crop protection application study.

In recent years, there has been rapid development of plant protection drones in China. Research related to plant protection drones in field crops has become relatively developed, while their application in fruit trees is still in its early stages. For example, research on parameters such as optimal operating height, optimal flight speed, and optimal spraying flow rate has been carried out on crops with small canopies, such as wheat, rice, and corn (Zheng et al., 2017; Kharim et al., 2019; Chen et al., 2020). In contrast, less research has been conducted on crops with large canopies such as fruit trees. Regarding whether the spray droplets from plant protection drones can penetrate the canopy of fruit trees? How can the optimal spraying volume be set to ensure better droplet deposition and suitable operational efficiency? What is the difference between plant protection drone spraying and backpack spraying deposition? These are the questions that bother everyone. Therefore, we conducted this study to address these questions. The flight control technology in orchard environments is challenging, and there is a high demand for it. In response to complex terrains such as hilly areas and basins, several drone companies in China have developed various product categories to ensure more uniform pesticide application using plant protection drones (Lan et al., 2022).To make plant protection drones meet the requirements of precision application operations, further research is needed on the effects of the number of droplets, spray coverage, and spray uniformity of representative plant protection drones. On the other hand, the Application Volume is an essential parameter for evaluating the effectiveness of plant protection drones. Due to the limited tank capacity and flight speed of plant protection drone sprayers, plant protection drones only use low-volume spraying during spraying (Lan and Chen, 2018). It is crucial to investigate whether plant protection drones’ low-volume spraying can achieve good droplet deposition and determine the optimal spray volume for plant protection drones’ spraying. Therefore, this paper conducted field experiments on mist droplet deposition distribution in citrus canopies at two spray volumes (60 L/ha and 120 L/ha) using two representative plant protection drones, XAG V40 and DJI T30, compared the results to mist droplet deposition with a knapsack electric sprayer (Knapsack Electric Sprayer, KES) at a spray volume of 2400 L/ha to investigate the spraying effect of plant protection drones in citrus orchards. The goal was to provide theoretical guidance and data support for the optimization of application parameters of plant protection drone orchard operation and pesticide application reduction and efficiency increase.

## 2 Materials and methods

### 2.1 Field plots

**Figure 1:**
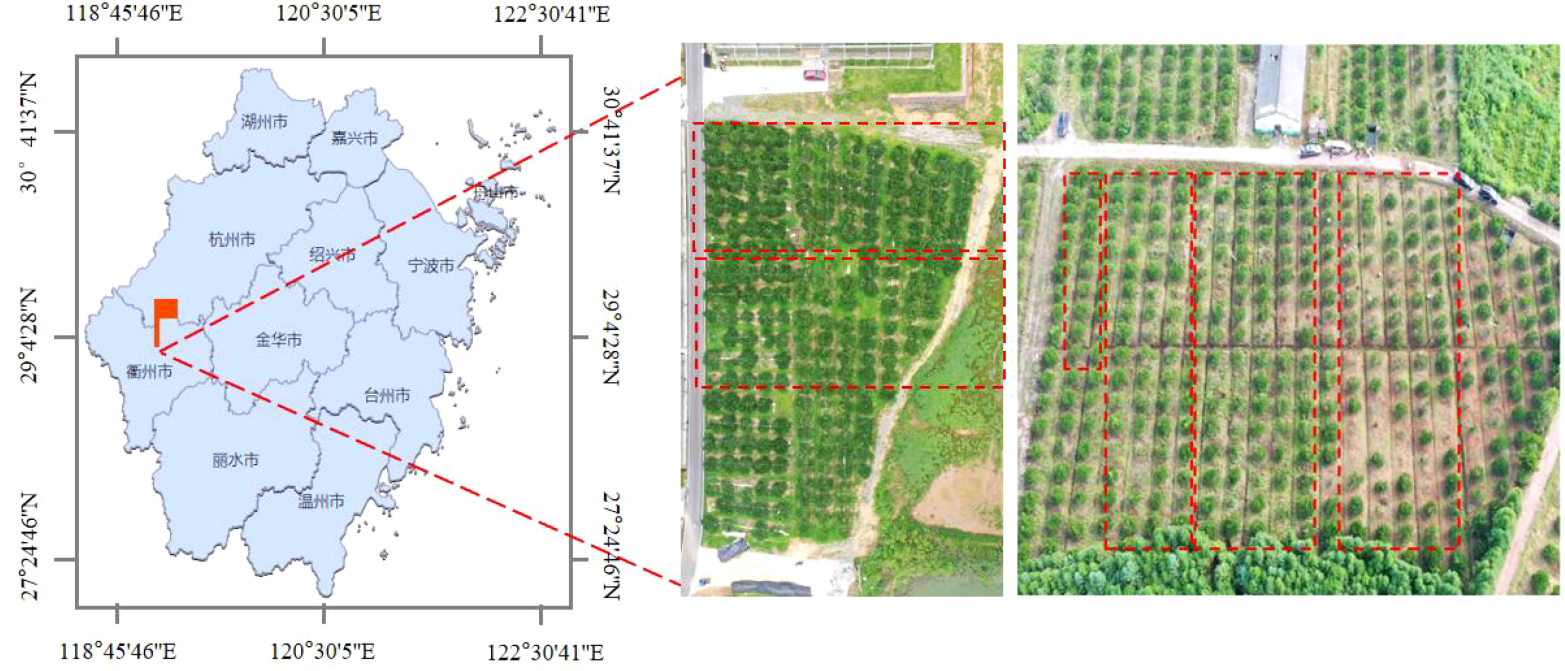
Experiment location.

The experiments were conducted in experimental fields located at the Tong Gong Township, Zhejiang Province (118°45’68″E, 28°58’52″N), China, in June 2021. The experiment material was citrus in the fruit-expanding stage. (2.6 ± 0.3) m high with a canopy diameter of (2.9 ± 0.3) m, planted at a density of 1523 trees/ha with a between-row spacing of (3.5 ± 0.5) m and a between-tree spacing of (2 ± 0.5) m (**Figure 1**). During the spray operation, the temperature was between 30.7°C and 32.6°C, relative humidity between 56.4% and 60.3%, and wind speeds between 0.0 and 1.3m/s. All meteorological parameters during the test period complied with the comparative measurement conditions of the ISO 22522 standard (International Organization for Standardization, 2007).

### 2.2 Plant protection drones and reference equipment selection

**Figure 2:**
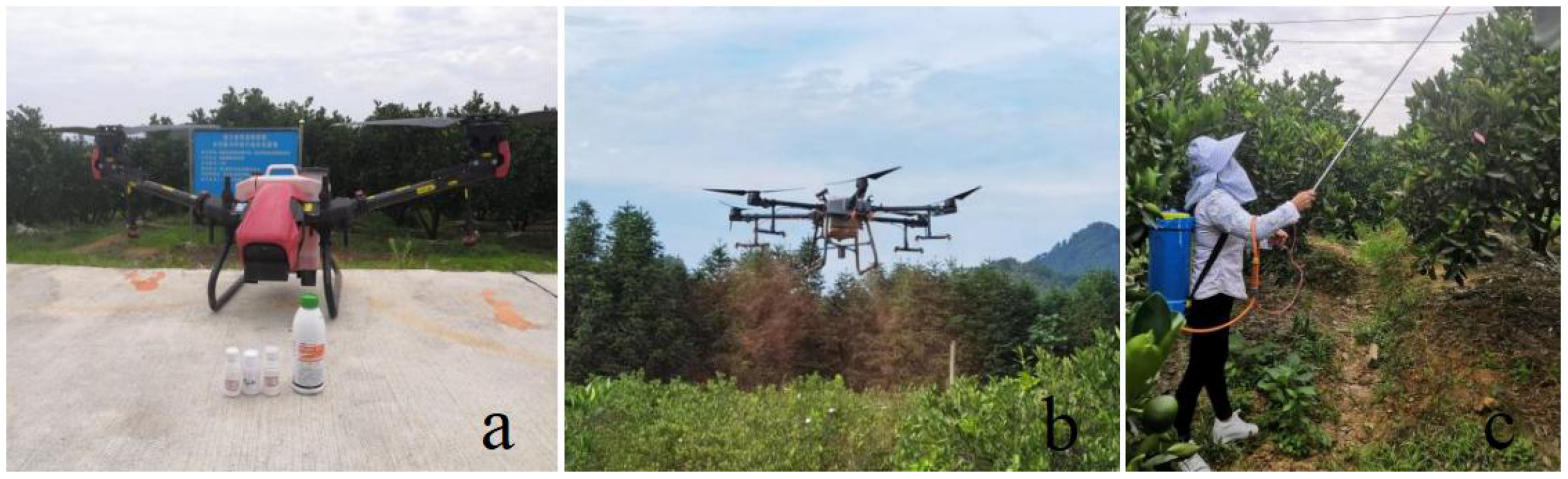
Spraying operations of the plant protection drones sprayer and KES in the citrus orchard: **(A)** XAG V40 two-rotor electric unmanned aerial vehicle. **(B)** DJI T30 six-rotor electric unmanned aerial vehicle. **(C)** Knapsack Electric Sprayer.

The spraying equipment was XAG V40 (XAG Co., Ltd., China) and DJI T30 (SZ DJI Technology Co., Ltd., China) (**Figure 2**). XAG V40 adopts a tilting dual-rotor structure, with rotor rotors on top of the two folding arms and a nozzle at the bottom, equipped with a 16 L tank, centrifugal nozzle, peristaltic pump, etc. It is equipped with a standard front dynamic radar, upward-looking radar, and ground-like radar, bringing a more comprehensive and delicate perception and obstacle avoidance capability. DJI T30 spray system is equipped with a horizontally opposed six-cylinder dual plunger pump design, a dual-channel electromagnetic flow meter error of ±2%, eight nozzles distributed downward on both sides of the fuselage, equipped with a spherical radar system, omnidirectional perception of the surrounding environment, to achieve intelligent around the obstacles and ground-like flight. The two representative plant protection drones, the XAG V40 and DJI T30 are officially given as having a maximum operating efficiency of up to 16 ha/h. The battery capacity of DJI T30 is 30Ah, and the battery capacity of XAG V40 is 20 Ah. One full charge allows for approximately 15 minutes of flight. The XAG V40 utilizes the XAG agricultural services software to plan flights and automatically carry out operations based on the preset routes. On the other hand, the DJI T30 uses the DJI agricultural software to plan flights and also performs operations automatically according to the preset routes. The detailed parameters of the equipment are shown in **Table 1**. Other test spraying equipment included a knapsack electric sprayer (3WBD-20-1, Taizhou Luqiao Huyue Sprayer Factory, China). The knapsack electric sprayer is equipped with an electric pump and atomizing nozzle, with a maximum spray width of 9 m, and a spray volume of 2400 L/ha was used for operation in this test. Other test materials included a scanner, a meteorological instrument, Kromekote cards, etc.

**Table 1:** Technical parameters for the plant protection drone sprayer.

Classification	XAG V40	DJI T30	KES
Dimensions/mm	2110mm×2127mm×555mm	2858mm×2685 mm×790mm	390mm×210mmx495mm
Rotor numbers	2	6	—
Nozzle number	2	16	1
Nozzle type	Centrifugal nozzle	Hydraulic nozzle	Hydraulic nozzle
Droplet size	130μm(10000 rpm)	105 μm(0.3 MPa)	175 μm(0.3 MPa)
Tank capacity/L	16 L	30 L	20 L
Spraying width/m	4m	4m	3m

### 2.3 Experimental design

The pesticide used in this experiment was Mancozeb 430 g/L SC. Before application, the Allura Red solution (80% purity, purchased from Beijing Oriental Care Trading Ltd., China) at a concentration of 5 g/L was added to the tank as a tracer. Before the experiment, set the experimental parameters such as flight height, flight speed, spraying width, droplet size, and application liquid volume according to the orchard situation. The spraying system automatically calculates the pump’s flow rate according to the parameters of flight speed, spraying width, and application liquid volume to ensure the accuracy of the application liquid volume. Two representative plant protection drones (XAG V40 and DJI T30) were tested for spray droplet deposition distribution in the citrus canopy, with a knapsack electric sprayer as a control. The application volume (APV) of the plant protection drones was set at 60 L/ha and 120 L/ha (**Table 2**). Other operating parameters of the plant protection drone application were set at flight height: 3 m, flight speed: 3 m/s, route spacing: 3.5 m, and Flow Rate:3.78L/min. During the trial, the plant protection drones flew along the tree rows and above the rows of citrus trees. The application volume of the knapsack electric sprayer was set at 2400 L/ha based on pesticide to water. The test personnel wear appropriate protective clothing, gloves, masks, and other personal protective equipment to ensure safety. Adjust the spray angle and pressure of the knapsack electric sprayer before the test. Carry the knapsack electric sprayer on the back to maintain the correct spraying distance and even moving speed. Spray different parts of the fruit tree, including the crown, branches, and leaves.

**Table 2:** Treatments designed for the field experiments.

Treatment	Sprayer	Application Volume(L/ha)
T1	XAG V40	60
T2	XAG V40	120
T3	DJI T30	60
T4	DJI T30	120
T5	KES	2400

**Figure 3:**
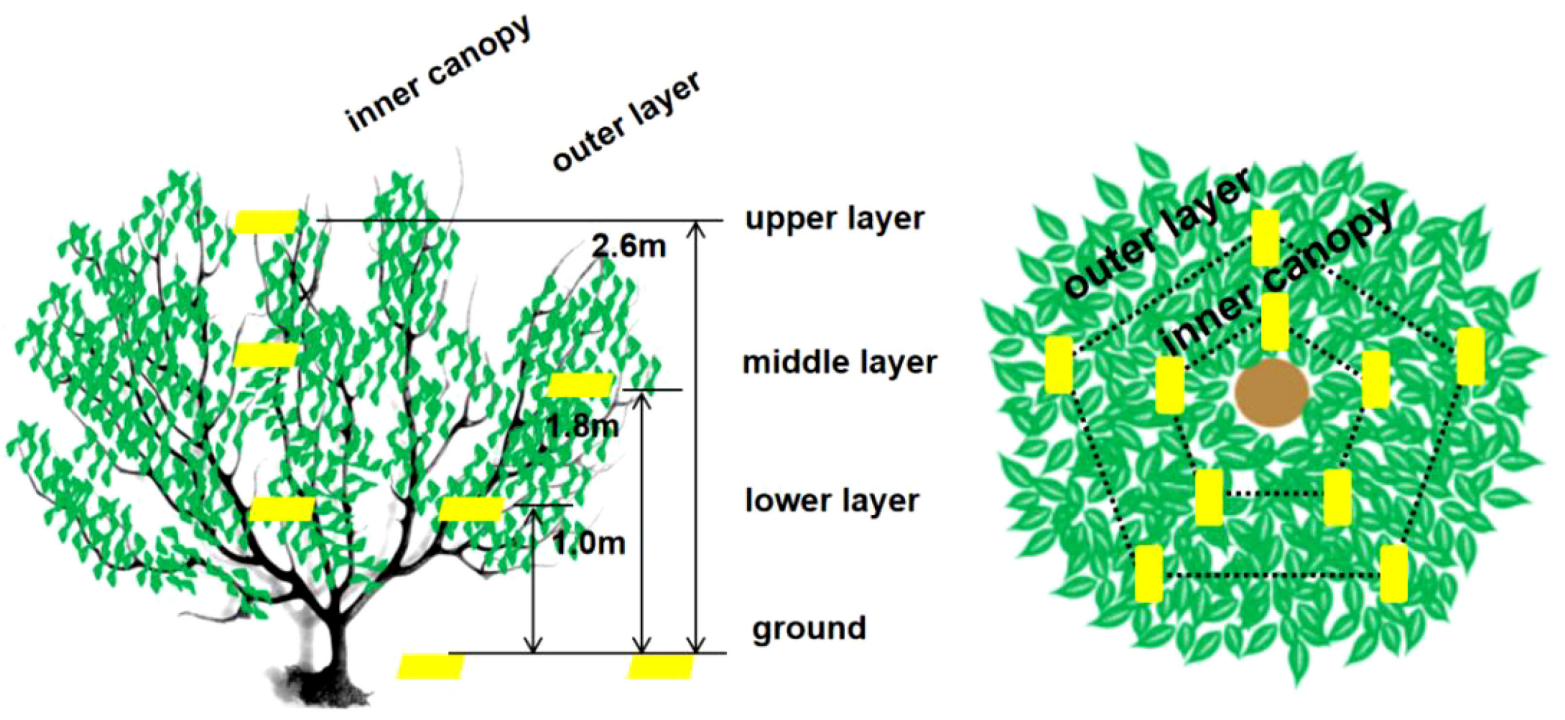
Sampler locations for assessing spray deposition in the target apple tree canopy and ground loss.

**Figure 4:**
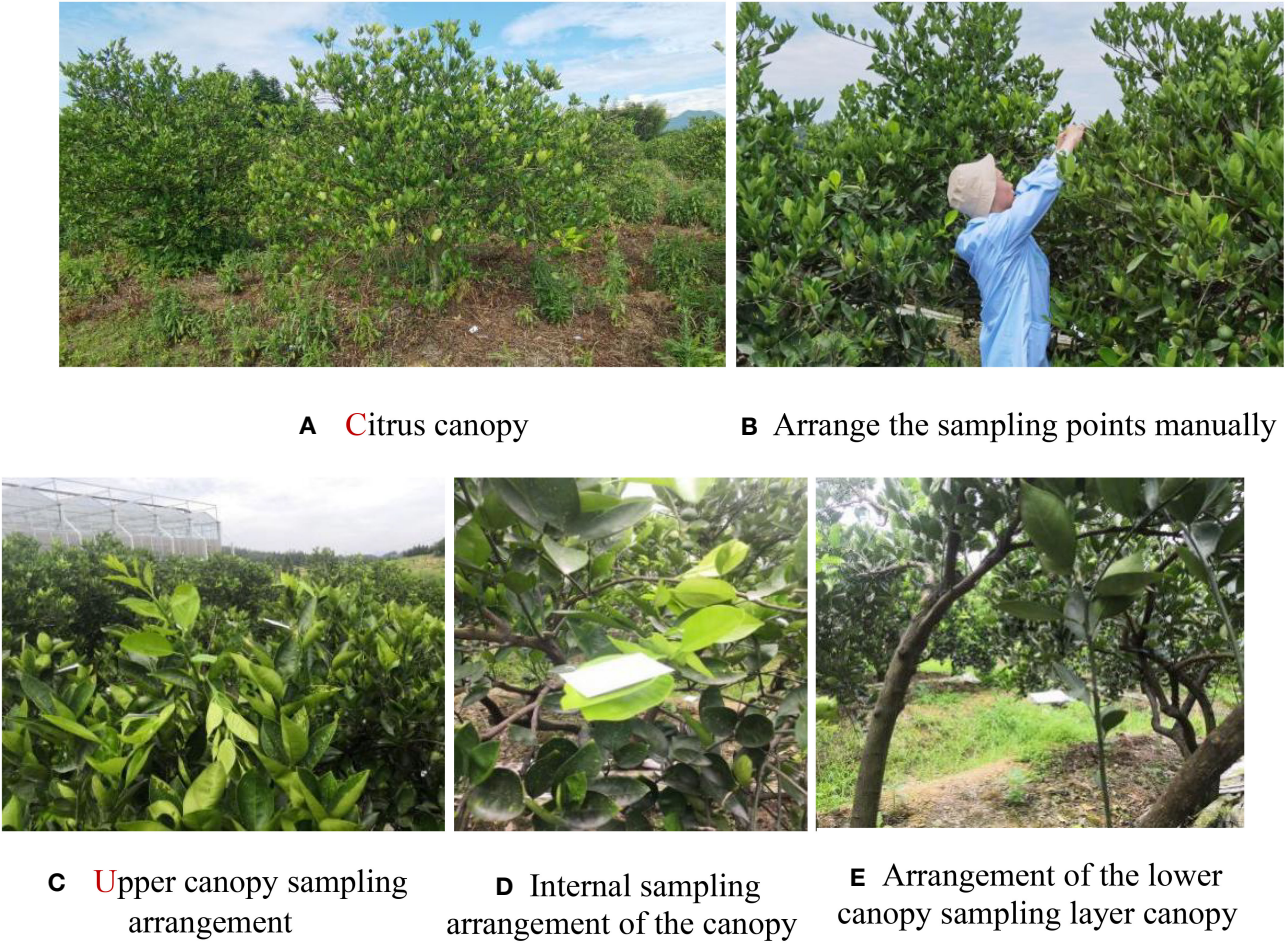
Materials of the field test. **(A)** Citrus canopy, **(B)** Arrange the sampling points manually, **(C)** Upper canopy sampling arrangement, **(D)** Internal sampling arrangement of the canopy, **(E)** Arrangement of the lower canopy sampling layer canopy.

In the sampling survey, each treatment group was divided into 5 plots in equal amounts according to the length of the treatment area, and 5 fruit trees of the same growth and shape were selected for each treatment group as multiple replications of the same treatment. For comparison with the reduced application by plant protection drones, one conventional control treatment area with the manual application by a knapsack electric sprayer was set up, and a blank control treatment area without pesticide application was designed. To avoid the effect of droplet drift between adjacent treatment zones, each treatment zone was used as a buffer zone within 10 m from the edge, and no droplet deposition data were collected within the buffer zone. Twenty-five sampling points were selected for each tree, and paper cards were fixed to the leaves with paper clips at each sampling point. The canopy of each citrus tree was divided into three layers, i.e., upper, middle, and lower, which included the upper layer (5 points), middle layer (inner canopy: 5 points; outer canopy: 5 points) and lower layer (inner canopy: 5 points; outer canopy: 5 points), and the deposition of droplets in different parts of the fruit tree canopy was collected (**Figures 3**, **4**). During the experiment, the pesticide droplets were not completely deposited in the canopy, and some droplets would pass through the canopy and deposit on the ground, causing soil contamination; therefore, 10 sampling points were arranged on the ground under each target citrus tree to collect the pesticides lost on the ground.

### 2.4 Sample and data analyses

After 30 minutes of each spraying test, dried WSPs were retrieved in sequence using disposable gloves. Put the WSPs into sealed bags marked according to the different treatments and add desiccant to prevent moisture from affecting the test results. Store the bags in a cool place and bring them back to the laboratory for analysis. All collected paper cards were scanned using a scanner, and the results were analyzed using the image processing software Deposit Scan (U.S. Department of Agriculture, Wooster, Ohio). Experimental results were processed using Excel 2019, and statistical analysis using SPSS 21.0. Duncan’s multiple comparisons test was used to analyze the significance of differences among various treatments.

Spray uniformity refers to the uniformity of spray droplet distribution on the target, and the uniformity of droplet distribution in the field has an important impact on pest control. To describe the uniformity of droplet distribution in the field, the Coefficient of Variation (CV) of the number of droplets in each sampling area was used to indicate the uniformity of droplet distribution for a set of data. The smaller the coefficient of variation, the smaller the variation in the data, i.e., the more uniform the distribution of droplet deposition, and the coefficient of variation was calculated as follows:


(1) $$\;\;CV=\frac{S}{\bar{X}}\times 100\%\;$$

(2) $$S=\sqrt{\;\sum_{i=1}^{n}\left(X_{i}-\bar{X}\right){\;}^{2}/\left(n-1\right)\;}$$

where S is the standard deviation of samples from the same test group; ${\text{X}}_{\text{i}}$ is the number of droplets or spray coverage at each sampling point; $\bar{\text{X}}$ is the mean value of samples from the same test group; and n is the number of sampling points in each test group.

## 3 Results

### 3.1 Analysis of the number of droplets and spray coverage

**Figure 5:**
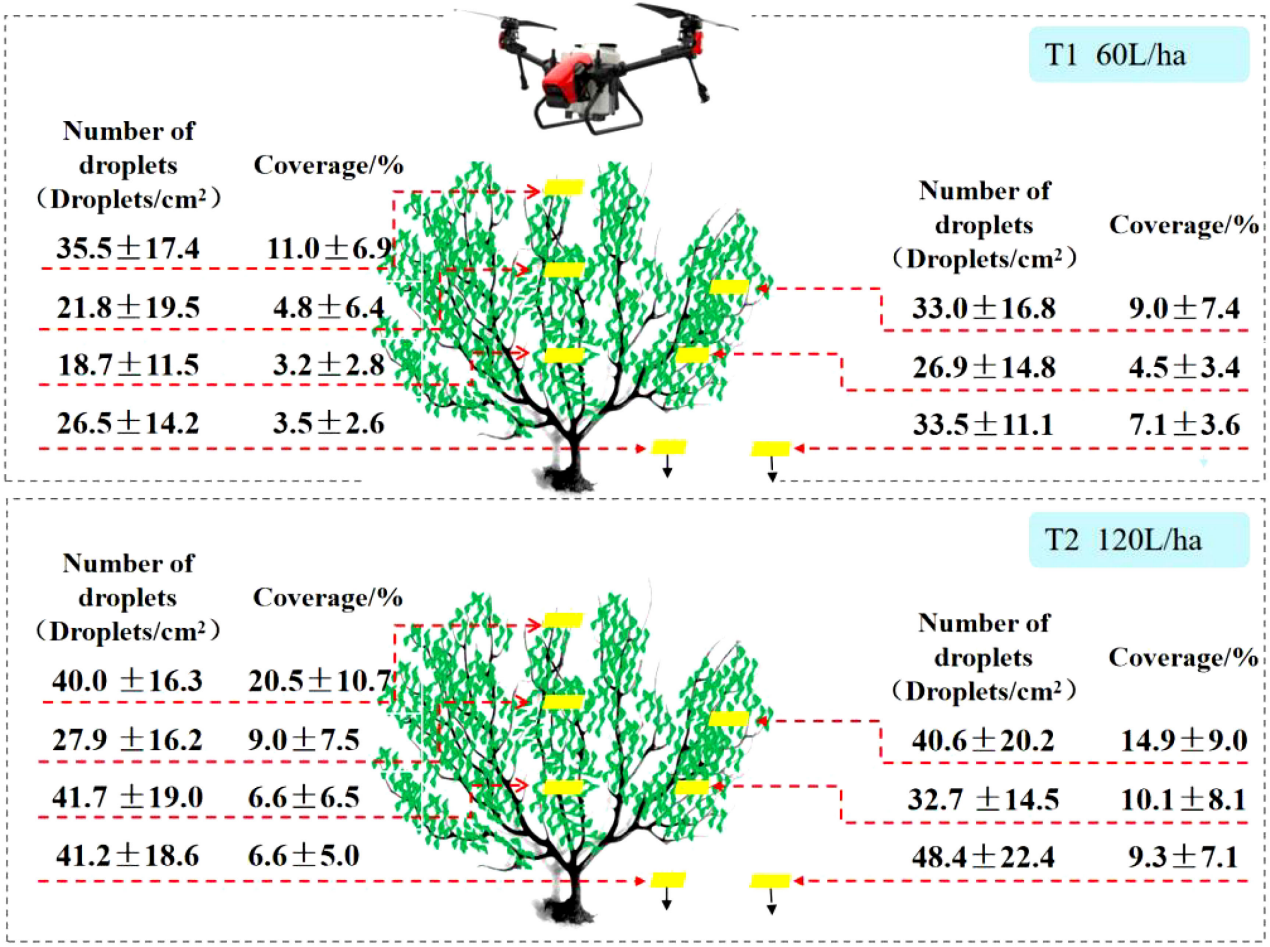
Different APV on the Number of droplets and spray coverage of XAG V40.

**Figure 5** presents the test results of the XAG V40 fruit tree application. From the perspective of the number of droplets, the results show that the number of droplets within the canopy of XAG V40 ranged from 18.7 droplets/cm^2^ to 35.5 droplets/cm^2^ at a spray volume of 60 L/ha. At a spray volume of 120 L/ha, the droplet density within the canopy ranged from 27.9 droplets/cm^2^ to 41.7 droplets/cm^2^. The percentage increase of the number of droplets in different parts of the canopy was 12.4% to 123.2% with an average increase of 34.5% compared to 60 L/ha. At a spray volume of 120 L/ha, the spray coverage in the canopy of XAG V40 ranged from 6.6% to 20.5%, while at 60 L/ha, it ranged from 3.2% to 11.0%. The increase in spray coverage in different parts of the canopy at a spray volume of 120 L/ha ranged from 65.7% to 127.6%, with an average increase of 87.8% compared to 60 L/ha.

Significant differences existed in the number of droplets and spray coverage of XAG V40 at different canopy sampling points of citrus trees. The results of spray coverage showed significant differences in spray coverage in the upper, middle, and lower layers of citrus trees. At a spray volume of 60 L/ha, the spray coverage at different canopy locations was in the following order: upper canopy > middle outer canopy > middle inner canopy > lower outer canopy > lower inner canopy. When the spray volume increased from 60 to 120 L/ha, the number of droplets showed the most significant change in the lower inner canopy, from 18.7 droplets/cm^2^ to 41.7 droplets/cm^2^, an increase of 123.2%. Additionally, the spray coverage in the lower canopy of the fruit trees was higher than that in the middle.

**Figure 6:**
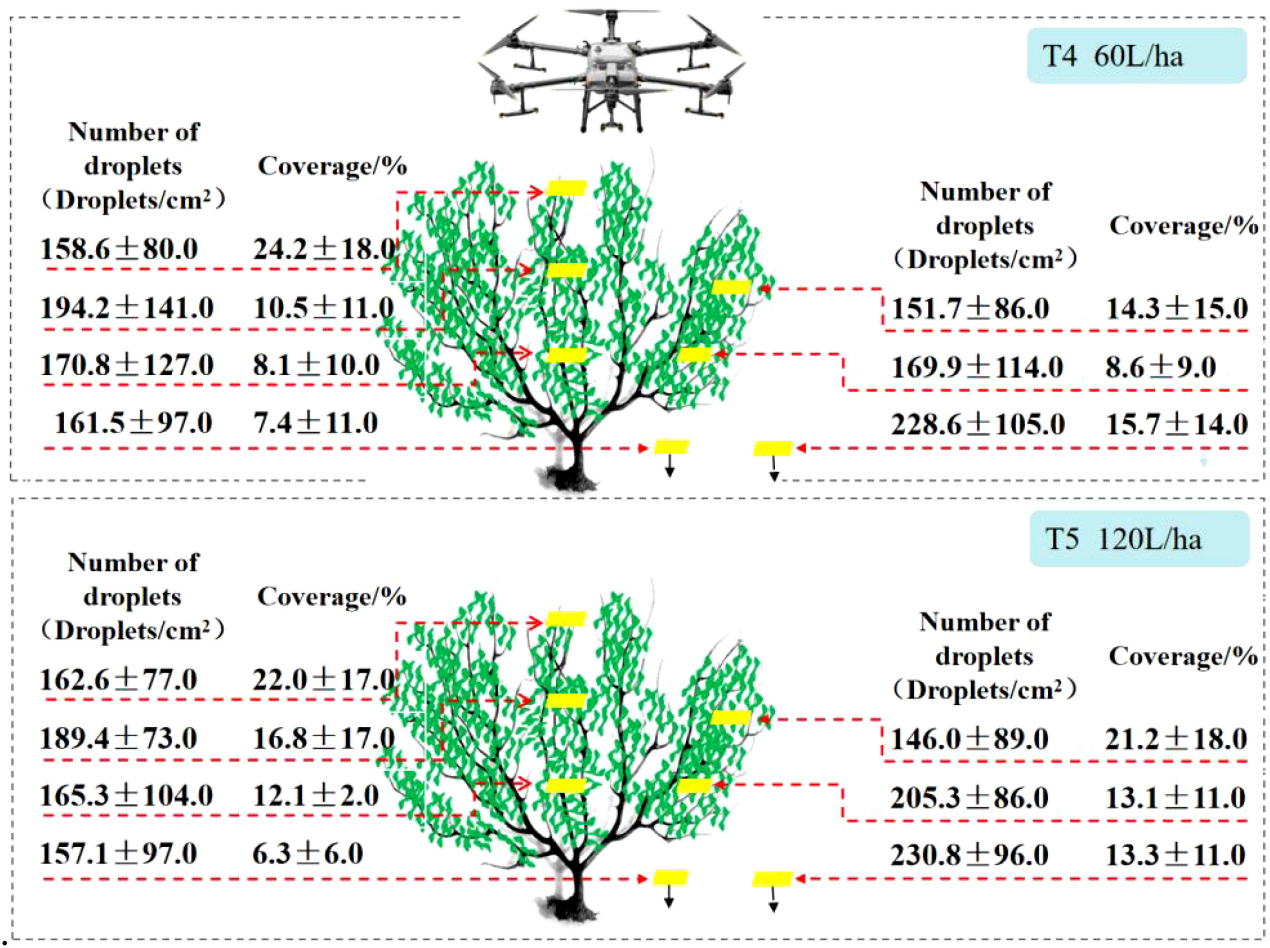
Different APV on the Number of droplets and spray coverage of DJI T30.

**Figure 6** shows the test results of the DJI T30 fruit tree application. It can be seen from the perspective of the number of droplets that at a spray volume of 60L/ha, the number of droplets within the canopy was 151.7 droplets/cm^2^ - 194.2 droplets/cm^2^, and at a spray volume of 120 L/ha, the number of droplets within the canopy was 146.0 droplets/cm^2^ - 205.3 droplets/cm^2^. However, there was no significant increase in the number of droplets at a spray volume of 120 L/ha, and the percentage variation in different parts of the canopy was -24.8% to 24.8%, with a spray coverage variation of -2.3%. From the perspective of spray coverage, this demonstrates that the DJI T30 had 8.6% - 24.2% spray coverage at a spray volume of 60 L/ha and 12.1% - 22.0% spray coverage at a spray volume of 120 L/ha. Compared with 60 L/ha, the spray coverage increased at a spray volume of 120 L/ha, especially in the lower and middle canopy, with a spray coverage increase of 52.5%.

In the DJI T30 fruit tree application test, there was no significant increase in the number of droplets in different canopies of citrus trees when the APV was increased from 60 to 120 L/ha. The change in spray coverage was more significant in the middle canopy, with an increase of 48.5% from 14.3% to 21.2% in the middle outer canopy and 60.1% from 10.5% to 16.8% in the middle inner canopy. Significant differences in the number of droplets were observed in different canopies of citrus trees. The spray coverage at different canopy locations at spraying rates of 60 L/ha and 120 L/ha followed this order: upper canopy > middle outer canopy > middle inner canopy > lower outer canopy > lower inner canopy.

The results showed that the spray volume of the plant protection drones affected the distribution of the number of droplets during the spraying operation, and the number of droplets and spray coverage varied at different spray volumes. When the APV increased from 60 to 120 L/ha, the number of droplets and spray coverage increased significantly, but this increase was not linearly proportional to the increase in APV.

**Figure 7:**
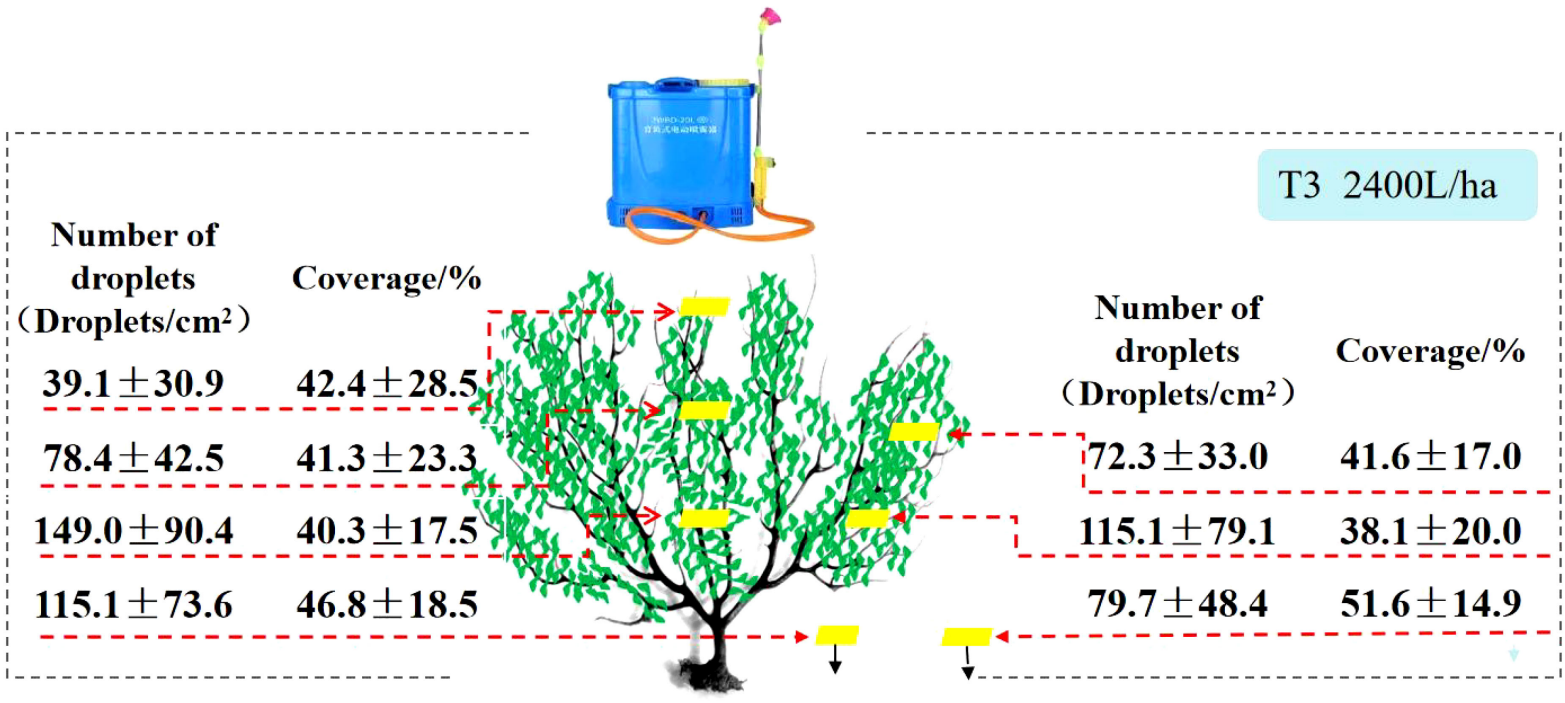
The Number of droplets and spray coverage of Knapsack Electric Sprayer.

**Figure 7** displays the experimental results of the knapsack electric sprayer fruit tree application. From the perspective of droplet deposition density, it can be seen that with a knapsack electric sprayer in 2400 L/ha spray liquid volume, droplet density within the canopy is 39.1 droplets/cm^2^-149.0 droplets/cm^2^; coverage within the canopy is 40.3% - 42.4%. The coverage within the canopy was 40.3% - 42.4%. The difference between the maximum and minimum values of **Figure 7** displays the experimental results of the knapsack electric sprayer fruit tree application. fruit tree applied droplet density was 109.9 droplets/cm^2^. The deposition in the lower canopy was much larger than in the upper canopy. The knapsack electric sprayer at 2400 L/ha of spray liquid volume had 48.3% higher upper canopy coverage than the XAG V40 at 120 L/ha and 51.9% higher upper canopy coverage than the DJI T30 at 120 L/ha. The knapsack electric sprayer had lower droplet density in the upper canopy than the two typical plant protection drones.

### 3.2 Analysis of droplet distribution uniformity

**Figure 8:**
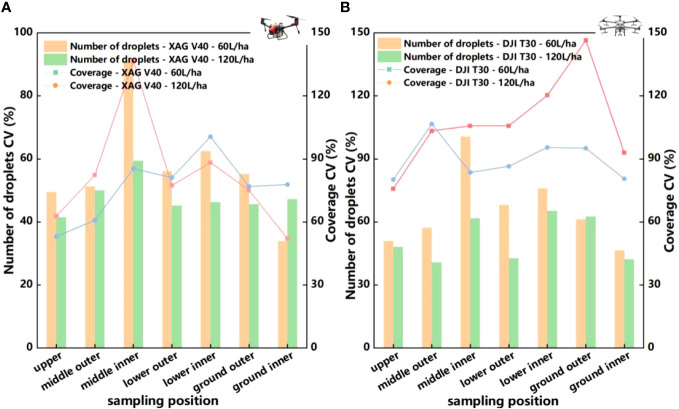
**(A)** Deposition distribution uniformity of XAG V40 two-rotor electric unmanned aerial vehicle in blade surface under 60 L/ha and 120 L/ha application volume. **(B)** Deposition distribution uniformity of DJI T30 six-rotor electric unmanned aerial vehicle in blade surface under 60L/ha and 120 L/ha application volume.

**Figure 8A** shows the analysis results of the coefficient of variation of the number of droplets and the coefficient of variation of spray coverage for XAG V40. As can be seen from the figure, the coefficient of variation of the number of droplets decreased to 2.6% - 39.3% when the spray volume increased from 60 to 120 L/ha. The mean coefficient of variation of the number of droplets within the canopy decreased by 22.0%. The mean coefficient of variation of intra-canopy spray coverage decreased by 14.9% when the spray volume was increased from 60 to 120 L/ha.

**Figure 8B** shows the results of the analysis of the coefficient of variation of the number of droplets and the coefficient of variation of spray coverage of DJI T30, and the results indicate that the increase of spraying volume helps to improve the uniformity of the number of droplets. When the spray volume increased to 120 L/ha, the coefficient of variation of the number of droplets decreased in all canopies, the mean coefficient of variation of the number of droplets decreased by 26.8% and the mean coefficient of variation of spray coverage decreased by 11.5%. The results showed that the uniformity of the number of droplets in the upper part of the canopy was better than that in the middle and lower parts of the canopy, whether it was 60 L/ha or increased to 120 L/ha. The deposition of droplets in the upper canopy of fruit trees is better than in the lower canopy, primarily because of the higher spraying position and smaller droplets used by plant protection drones, making it difficult for the droplets to penetrate the dense foliage. Although increasing the downdraft can enhance the deposition of droplets in some lower positions of the canopy, it can also cause the canopy to collapse, resulting in poorer uniformity of droplet distribution in the lower canopy. As shown in **Figure 8**, the change in the middle and lower canopy is most noticeable after increasing the application volume. This indicates that increasing the application volume enhances droplet deposition in different canopy layers and improves penetration, thereby influencing the uniformity of droplet deposition in the middle and lower layers. Suitable working parameters can be selected for different spraying requirements and machine types.

From the above analysis, it is clear that an increase in spray volume also improves spray uniformity. Within the canopy, the coefficient of variation of the number of droplets in the central inner part showed the greatest variation, decreasing by 34.9%. This indicates that increasing the spray volume increased droplet penetration and made it easier to penetrate the canopy. The humidity inside the crop canopy is generally higher, and some diseases occur from inside the canopy. Therefore, less distribution of droplets inside the canopy will reduce the spraying effect. Therefore, the use of plant protection drones for the application of chemicals, for the control of pests and diseases mainly in the inner canopy of citrus, increasing the amount of spray liquid can improve the coverage and deposition uniformity to achieve better prevention effect, but the specific impact also needs to take into account the effect of canopy size, resistance and wind and other factors. The effect of other factors such as droplet size and leaf density on spraying effectiveness should also be noted.

### 3.3 Comparative analysis of deposition on the citrus

**Figure 9:**
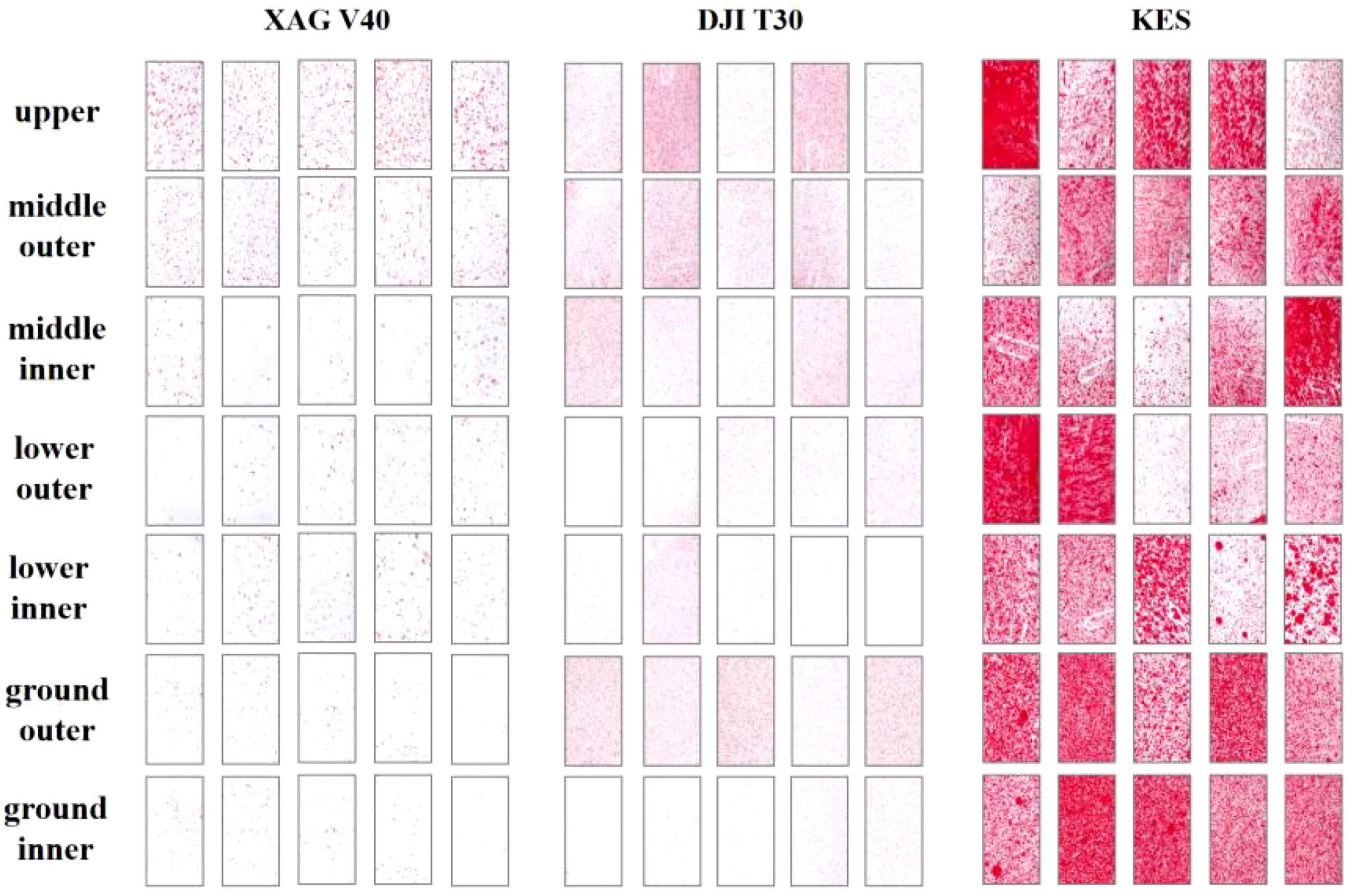
Visual diagram of the deposition of spray droplets in citrus orchards by different plant protection machinery.

A visualization of the droplet deposition distribution for the XAG V40, DJI T30, and knapsack electric sprayer segments (**Figure 9**) shows that the droplet particle size of the plant protection drones is on average smaller than that of the knapsack electric sprayer and is better droplet uniformity. The knapsack electric sprayer spray may have droplets that will converge into larger droplets.

In the liquid spray volume application test between two plant protection drones, XAG V40, and DJI T30, the DJI T30 had finer droplet particle size, 4.9 - 8.1 times higher number of droplets, and 1.1-2.5 times higher coverage compared with XAG V40. It indicates that under the wind field and operating parameters conditions of this test plant protection drone, the fruit tree nozzle used by DJI T30 is beneficial to deliver the liquid to each canopy layer. The changes in the number of droplets and coverage of XAG V40 were more significant when the spray volume was increased from 60 L/ha to 120 L/ha, indicating that the spray volume could be increased to increase the droplet deposition distribution as well as the droplet penetration.

**Figure 10:**
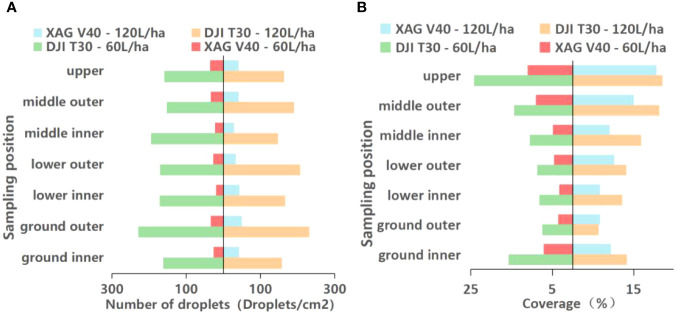
**(A)** Number of droplets of XAG V40 and DJI T30 on blade surface. **(B)** Coverage rate of XAG V40 and DJI T30 in the blade surface.

The rotor wind field of plant protection drones facilitates the penetration of droplets into the canopy, but it also tends to cause uneven droplet distribution. As shown in the **Table 3**, the uniformity of droplet distribution within the canopy is similar between XAG V40 and DJI T30, and the coefficient of variation of the number of droplets is between 50% and 100%. The increase in APV from 60 to 120 L/ha and the increase in the coefficient of variation of the number of droplets between XAG V40 and DJI T30 indicates that the increase in spray volume helps to improve the uniformity of spraying. At the same time, the statistics show that XAG V40 has relatively high uniformity despite its lower number of droplets. This indicates that the wind field under the rotor of the plant protection drones is the main factor affecting the distribution pattern of droplet deposition (**Figure 10**). A weak or disturbed wind field is very likely to make it unable to penetrate the crop canopy, and the droplets cannot reach the lower and inner layers of the canopy, resulting in uneven distribution among the canopies and affecting the control effect. The wind field generated by different types of plant protection drones differs due to different aerodynamic principles and significant structural differences. The wind field generated by multi-rotor plant protection drones during operation is the result of the interaction of airflow generated by multiple rotors (Chen et al., 2017), which differs greatly from the wind field and downforce airflow generated by dual-rotor plant protection drones, resulting in significant differences in the deposition distribution of droplets in the crop canopy between different types of plant protection drones.

**Table 3:** Number of droplets coefficient variation and spray coverage coefficient variation of XAG V40 and DJI T30.

The type of drone of application volume		Coefficient of variation of number of droplets (%)	Coefficient of variation of spray coverage(%)
XAG V40	60L/ha	62.1	89.5
	120L/ha	48.5	76.2
Ratio		-22.0	-14.9
DJI T30	60L/ha	70.6	102.2
	120L/ha	51.7	90.5
Ratio		-26.8	-11.5

**Figure 11:**
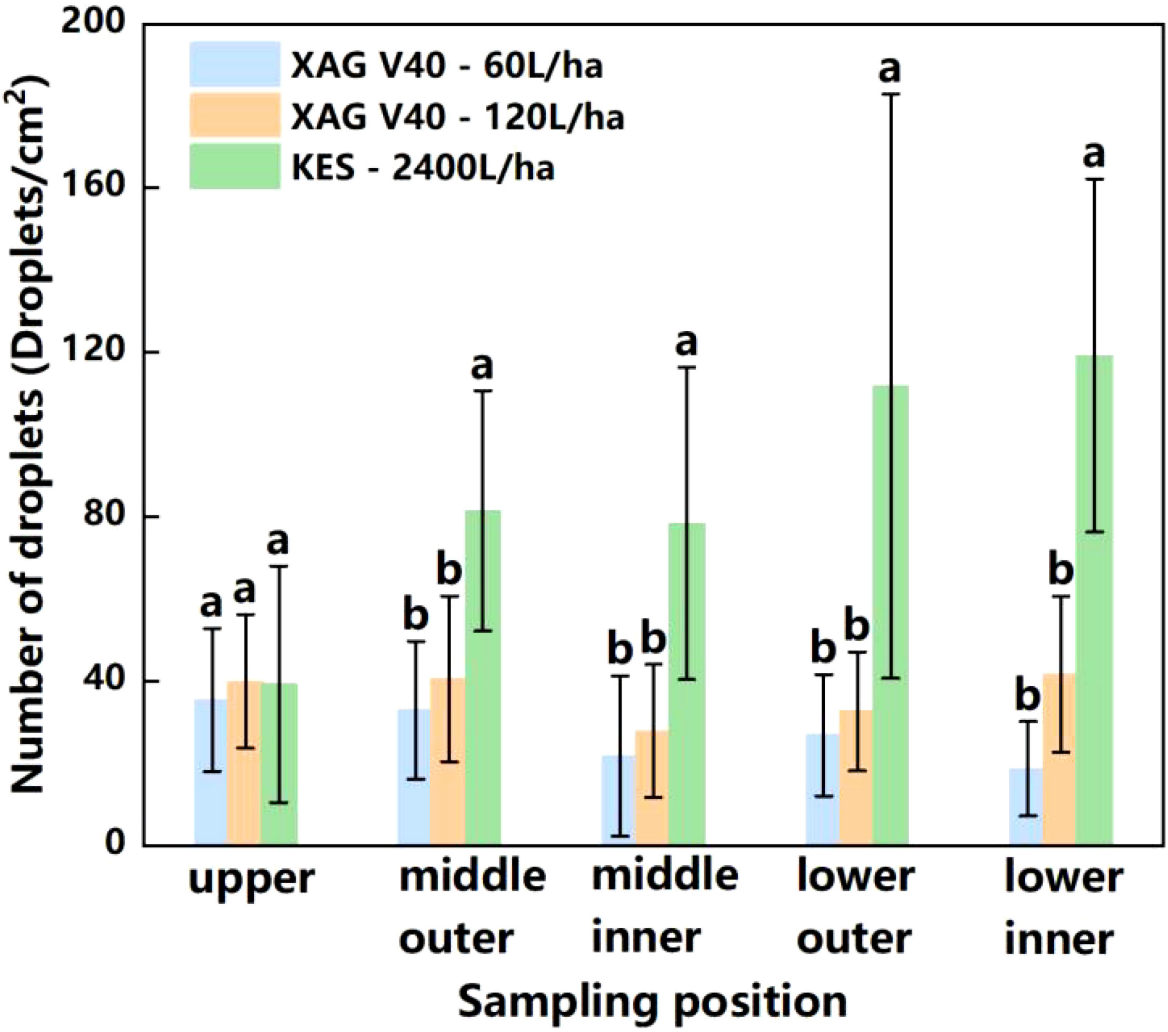
Number of droplets of XAG V40 and Knapsack Electric Sprayer. (a, b express that there are significant differences between different spray methods and spray volume. P<0.05).

Compared to the plant protection drones, the knapsack electric sprayer applied 20 times more liquid. It increased the number of droplets by 100.7% - 241.9% at different locations in the middle and lower part of the crop canopy, with the highest growth rate of 241.9% in the lower outer layer (**Figure 11**). Due to the high spray volume of the knapsack electric sprayer, the inner canopy leaf droplet density was higher compared to the plant protection drones, but the upper canopy was lower than the plant protection drones. The deposition of the knapsack electric sprayer treatment group was concentrated on the outer part of the middle and lower canopy, indicating that under the present test conditions, the pesticide droplets from the knapsack electric sprayer still cannot penetrate the upper canopy, while the droplets produced by the plant protection drones are conducive to deposition in the upper canopy, which can improve the pest and disease control effect in the upper canopy. The knapsack electric sprayer showed a decreasing trend from bottom to top, while the plant protection drones were relatively more uniform in the upper, middle, and lower canopy layers of the fruit tree canopy. This is because the low-volume spraying of plant protection drones can ensure the maximum increase of droplet deposition distribution with small droplet particle size, whereas the traditional manual spraying results in larger droplet particle size and poor penetration. In addition, the limitation of the number of droplets and the spray coverage in the citrus canopy and weed inter-distribution further affects the spraying efficiency.

### 3.4 Analysis of loss in ground

**Figure 12:**
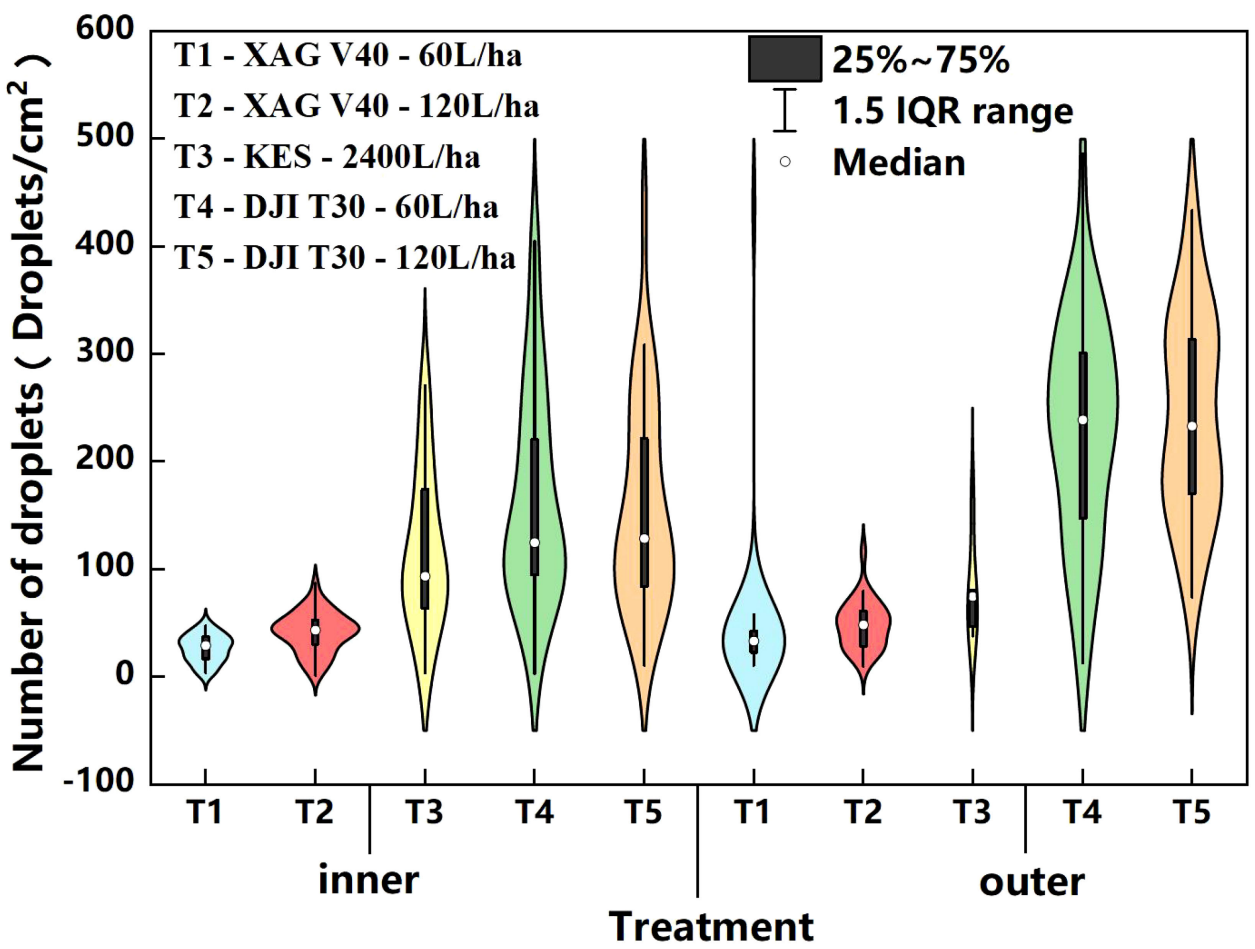
Ground loss for the five treatment groups.

In actual operation, due to the complex distribution of the overall leaf inclination angle of citrus tree leaves, the large droplets generated by plant protection equipment spraying are prone to run off from the leaves and cause pesticide loss. The results of the droplet deposition on the ground sampling area of XAG V40, DJI T30, and the knapsack electric sprayer are shown in **Figure 12**.

In the XAG V40 application test, the number of droplets was higher at locations where the ground was unobstructed by branches and leaves. When the spray volume was increased, the droplet density increased at all ground sampling points. When the APV increased from 60 L/ha to 120 L/ha, there was a 58.6% increase in droplet density at ground locations with branch and leaf shading. The number of droplets increased by 44.3% at the ground level without branch and leaf shading. In the DJI T30 application test, the ground loss was insignificant when the spray volume was increased. Increasing the APV from 60 L/ha to 120 L/ha reduced the number of droplets by 2.7% at the ground level with branch and leaf shading and increased it by 0.9% at the ground level without shading.

Compared to the plant protection drones, the number of droplets from the knapsack electric sprayer on the ground in both shaded and unshaded positions was higher than that of XAG V40 but lower than that of DJI T30. The number of droplets in the unshaded position on the ground of the knapsack electric sprayer was lower than that of the shaded position on the ground. Even though the number of droplets in the shaded position on the ground was higher than that inside the lower canopy, the reason is that the knapsack electric sprayer nozzle has a high flow rate, and the atomization effect is poor, causing droplets to form large droplets or even spray directly to the ground, resulting in poor spraying effectiveness. Moreover, due to the presence of branches inside the canopy, the absorption of the droplets is weaker than that of the leaves, and droplets fall to the ground above the inter-plant gap area.

## 4 Discussion

Pesticide application technology plays a vital role in orchard production and is essential for improving citrus quality. Plant protection drones enable independent spraying operations without being subject to crop growth patterns and terrain restrictions, which significantly improves application efficiency and precision. However, the main concern with using plant protection drones is whether low-volume application leads to insufficient deposition and affects the effectiveness of control. Furthermore, not all sprayed solute ions are deposited on the target, and the off-target portion can be lost to the environment, causing contamination. These benefits can only be realized if a full assessment of spray droplet deposition effects is conducted. This study examined two typical plant protection drones, XAG V40, and DJI T30, for their reduced application in citrus orchards. The results showed that the plant protection drones deposited droplets with a density of approximately greater than 25 droplets/cm^2^ in each longitudinal and lateral layer of fruit trees, meeting the plant protection requirements of orchards (Administration of Quality Supervision, Inspection and Quarantine of the People’s Republic of China, 2008). The use of different spray volumes by plant protection drones significantly affected leaf coverage, droplet density, and spray uniformity (Wandkar et al., 2018; Gil et al., 2021). The DJI T30 achieves better deposition effects compared to a manually operated knapsack electric sprayer with a spray volume of 2400 L/ha by using a spray volume of 60 L/ha. Citrus crops require a lower droplet density when lightly infected or when applying systemic agents. This allows the use of plant protection drones with a lower application volume, which can achieve a droplet density of approximately 25 droplets/cm^2^, meeting the requirements for pest control. This study found that increasing the spray volume of the XAG V40 from 60 L/ha to 120 L/ha resulted in an average increase of 34.5% in droplet density in different canopy parts. Therefore, in the case of severe disease infection in citrus crops, increasing the application volume can improve droplet deposition effects and meet the requirements for pest control.

The use of different spray volumes by plant protection drones has a significant impact on leaf coverage and droplet density. Previous studies have shown that droplet density increases with increasing spray volume, which is consistent with our findings that both droplet density and coverage increased when the spray volume of the plant protection drone was increased from 60 L/ha to 120 L/ha in the trial. However, in a controlled trial with manual application from a knapsack electric sprayer, it was found that the knapsack electric sprayer using 20 times higher spray liquid volume than that of the plant protection drone increased droplet density at different locations in the lower and middle parts of the crop canopy by only 100.7% - 241.9%. The upper spray liquid effect of the knapsack electric sprayer was significantly lower than that of the plant protection drone. This is due to the lack of droplet-assisted diffusion device in the knapsack electric sprayer, and it takes some time for the liquid to reach the surface of the crop leaves from the nozzle, resulting in part of the liquid evaporating and drifting away, and reducing the surface coverage of the leaves (Hussain et al., 2022a; Hussain et al., 2022b). At the same time, the spraying height of the knapsack electric sprayer in the actual application process depends on the operator, and the deposition effect is also closely related to the operator’s spraying method.

Increasing the amount of liquid spray can improve the uniformity and penetration of spray droplets applied by plant protection drones. Theoretically, the number of droplets generated by plant protection drone operations in the citrus canopy should gradually decrease from the upper to the lower layers. However, the results of this experiment did not show a gradual weakening trend from top to bottom, and the number of droplets in the lower or middle layer of the fruit tree canopy was slightly higher than that in the upper layer. This may be mainly because, in practice, plant protection drones can penetrate droplets better into the canopy. Guo et al. (2020) verified through experiments that the downward spiral of plant protection drones downwash airflow can blow open the fruit tree canopy and improve the penetration of droplets. The downward airflow generated by the rotor also helps increase the adhesion and penetration of droplets on the crop, making the plant protection drones relatively more uniform in the different canopy layers of fruit trees at the top, middle, and bottom. The Miranda-Fuentes (Praat et al., 2000) trial showed that an increase in application rate increased average deposition and coverage but decreased application efficiency, spray penetration, and deposition uniformity. However, in this trial, comparing the deposition uniformity of the XAG V40 plant protection drone and the DJI T30 plant protection drone, the results showed that the uniformity of droplet distribution within the canopy was essentially similar between the XAG V40 plant protection drone and the DJI T30 plant protection drone, with coefficients of variation for droplet deposition in the range of 50% - 100%. the APV increased from 60 L/ha to 120 L/ha, the coefficient of variation of fog droplet density increased for the XAG V40 plant protection drone and DJI T30 plant protection drone, suggesting that the increase in spray volume also contributed to improved spray uniformity. The uniformity of fog droplet deposition on each layer of the fruit tree canopy was poor in this experiment. The reason for this phenomenon may be that the wind field below the rotor of the plant protection UAV is too strong, causing the upper branches of the fruit tree plants to tilt in all directions. Most of the droplets reach the middle and lower parts of the fruit tree plants with the wind field, and fewer droplets are deposited around the upper layers of the canopy, and the tilted branches affect the deposition of droplets on the leaves of the fruit trees so that the amount of droplet deposition on the leaves in the direction of the inverted direction is less.

Spray volume and sprayer type affect spray distribution and off-target losses in the canopy. Both liquid spray volume and sprayer type affect spray distribution and off-target losses in the canopy. The results of this experiment showed that when the liquid spray volume was increased from 60 L/ha to 120 L/ha, the droplet density at the ground sampling point increased with the elevated liquid spray volume. Compared with XAG V40 and DJI T30 plant protection UAVs, the loss of knapsack electric sprayer on the ground was higher than the XAG V40 plant protection UAV and lower than the DJI T30 plant protection UAV. Wise et al (Zhang et al., 2016). sprayed with different liquid spray volumes, and the loss of liquid solution was the most significant at the highest liquid spray volume. However, the effect of this liquid loss also depended on the canopy size, with a gradual decrease in fog droplet coverage from top to bottom for the different canopies tested by plant protection drones, which was mainly related to the structural state of the fruit tree form (Ferguson et al., 2015). Zhang Pan et al (Miranda-Fuentes et al., 2015). found that during production management, open citrus trees were suitable for plant protection drone spraying, but fluctuations and large dispersions showed poor uniformity, and these consequences may be related to the pruning of the tree structure. Maintaining a high standard and uniformity of the open canopy is difficult, and this still needs improvement. Combined with the plant protection UAV spraying test in this study, increasing the amount of liquid spray has a significant effect on improving uniformity, and how to obtain the optimal parameters for plant protection UAV spraying and reducing the loss of liquid solution will be a key issue that will continue to be of concern for the future application of plant protection UAVs.

Many other factors affect the effect of fog droplet deposition by plant protection drones, and more comprehensive field trial studies are needed. Including nozzle type, nozzle installation position, and angle, the interface of spray width between multiple nozzles, the use of parameters (such as spray pressure, flight speed, etc.), and the natural environmental conditions during spraying. In addition, the rotor wind and side wind in aerial applications can also easily cause uneven droplet distribution. Researchers have conducted comprehensive indoor performance tests on nozzles. Ferguson et al (Wise et al., 2010). compared the droplet size distribution of 21 spray drift reduction nozzles in a wind tunnel for three liquids with different dynamic surface tensions. The nozzle types classified as homogeneous in the study were XR, ABJ 11002, AITTJ60, and AIXR 110015, among others. The average CV for each nozzle type was equal to or less than 4%, and most of the tested nozzles were homogeneous in the nozzle cell. However, the uniformity of droplet distribution at different operating parameters needs to be analyzed for specific aerial application equipment.

Improving deposition also requires the right amount of spray liquid in conjunction with other operating parameters and minimizing drift. Experts suggest that additives can improve deposition. Gimenes et al. (2013)found that the use of spraying aids has great potential to improve the uniformity of pesticide spraying, increase spray coverage, and reduce the amount of pesticide applied. Guo Shuang et al (Guo et al., 2022). found that the use of additives significantly increased the particle size of droplets and reduced the proportion of small droplets in the experiment of evaluating additives in the South Fruit Pear Garden, which can effectively reduce the risk of droplet drift and help to improve the pesticide utilization rate. In addition, studies have shown that flight mode, flight altitude, and side wind speed all affect plant protection UAV spray droplets (Qin et al., 2016; Wang et al., 2016), because the downward rotating airflow wind field affects the droplet motion during plant protection UAV operations, and changes in altitude alter the uniformity of droplet distribution (Wang et al., 2022). Therefore, it is necessary to fully study the effects of the body structure and flight parameters of plant protection UAVs on droplet deposition, explore reasonable use techniques in the field, and provide data support for future use, to minimize the adverse effects of plant protection UAV operations and better utilize the advantages of plant protection UAVs in orchard control.

## 5 Conclusion

Plant protection drones are a powerful tool for improving fruit tree spraying operations and enhancing the quality of droplet deposition. Due to factors such as the rotor wind field of the plant protection drones, the unique tree structure of different fruit trees, the terrain environment, and other influencing factors, it is necessary to optimize the operating parameters of the plant protection drones to ensure the effective distribution of droplets during aerial spraying operations in the fruit tree canopy. In this paper, the effects of XAG V40 and DJI T30 on droplet deposition distribution on citrus trees at 60 L/ha and 120 L/ha spray volume were compared experimentally, and the droplet deposition distribution characteristics of the knapsack electric sprayer on citrus trees were also analyzed, along with ground loss for each experiment. Extensive field experimental results showed that the pesticide droplets still cannot penetrate the upper canopy when applied by a knapsack electric sprayer, while the droplets produced by plant protection drones are conducive to deposition in the upper part of the canopy. Increasing the amount of applied liquid is more significant for the deposition of droplets in the upper part of the citrus canopy, which helps the utilization of pesticides. Therefore, using plant protection drones to control pests and diseases in the middle and upper part of the citrus canopy and increasing the amount of liquid applied will achieve better control effectiveness. Further research should seek to assess the impact of additional fungicides and insecticides to ascertain the relevance of the conclusions since the data are only based on a small spectrum of active ingredients. It is worth noting that the wind field under the plant protection drone rotor can increase the penetration of droplets among crop plants, and the strength of the rotor wind field varies among different types of plant protection drones. Thus, further research on the rotor wind field of plant protection drones is needed to improve the spraying performance of plant protection drones. Additionally, some pests and diseases attached to the abaxial surface of leaves should be considered in future studies to further investigate the deposition effect on the abaxial surface of leaves to better understand the performance of fog droplet deposition.

## Data availability statement

The original contributions presented in the study are included in the article/supplementary material. Further inquiries can be directed to the corresponding authors.

## Author contributions

YL: Methodology, Resources, Writing – review & editing. YY: writing – Original draft, Validation. GW: Formal analysis, Validation, Writing – original draft. MH: Investigation, Writing – review & editing. HW: Investigation, Writing – review & editing. XY: Investigation, Writing – review & editing. CFS: Data curation, Writing – review & editing. BW: Data curation, Writing – review & editing. CCS: Formal analysis, Validation, Writing – review & editing.
